# PeTTSy: a computational tool for perturbation analysis of complex systems biology models

**DOI:** 10.1186/s12859-016-0972-2

**Published:** 2016-03-10

**Authors:** Mirela Domijan, Paul E. Brown, Boris V. Shulgin, David A. Rand

**Affiliations:** Current address: The Sainsbury Laboratory, University of Cambridge, Bateman Street, Cambridge, CB2 1LR UK; Warwick Systems Biology Centre, University of Warwick, Gibbet Hill Road, Coventry, CV4 7AL UK; M&S Decisions LLC, Black Swan villa, Naryshkinskaya al., Moscow, 125167 Russia

**Keywords:** Perturbation theory, Dynamical systems, Mathematical models, Gene regulatory networks, Signalling systems

## Abstract

**Background:**

Over the last decade sensitivity analysis techniques have been shown to be very useful to analyse complex and high dimensional Systems Biology models. However, many of the currently available toolboxes have either used parameter sampling, been focused on a restricted set of model observables of interest, studied optimisation of a objective function, or have not dealt with multiple simultaneous model parameter changes where the changes can be permanent or temporary.

**Results:**

Here we introduce our new, freely downloadable toolbox, PeTTSy (*Pe*rturbation *T*heory *T*oolbox for *Sy*stems). PeTTSy is a package for MATLAB which implements a wide array of techniques for the perturbation theory and sensitivity analysis of large and complex ordinary differential equation (ODE) based models. PeTTSy is a comprehensive modelling framework that introduces a number of new approaches and that fully addresses analysis of oscillatory systems. It examines sensitivity analysis of the models to perturbations of parameters, where the perturbation timing, strength, length and overall shape can be controlled by the user. This can be done in a system-global setting, namely, the user can determine how many parameters to perturb, by how much and for how long. PeTTSy also offers the user the ability to explore the effect of the parameter perturbations on many different types of outputs: period, phase (timing of peak) and model solutions. PeTTSy can be employed on a wide range of mathematical models including free-running and forced oscillators and signalling systems.

To enable experimental optimisation using the Fisher Information Matrix it efficiently allows one to combine multiple variants of a model (i.e. a model with multiple experimental conditions) in order to determine the value of new experiments. It is especially useful in the analysis of large and complex models involving many variables and parameters.

**Conclusions:**

PeTTSy is a comprehensive tool for analysing large and complex models of regulatory and signalling systems. It allows for simulation and analysis of models under a variety of environmental conditions and for experimental optimisation of complex combined experiments. With its unique set of tools it makes a valuable addition to the current library of sensitivity analysis toolboxes. We believe that this software will be of great use to the wider biological, systems biology and modelling communities.

**Electronic supplementary material:**

The online version of this article (doi:10.1186/s12859-016-0972-2) contains supplementary material, which is available to authorized users.

## Background

There is a rapidly increasing number of complex, high dimensional deterministic models in Systems Biology and these play a crucial role in gaining an understanding of important biological systems that would be impossible to achieve using lab-based approaches alone. Tools that can be used in a systems biology iterative cycle to enable the development and analysis of models and their fitting to data are becoming increasingly important.

Sensitivity analysis is an important approach that has been successfully employed to do the above, but it is just one part of dynamical systems perturbation theory [1, 2]. This extensive theory enables one to probe the behaviour of dynamical systems locally in parameter space. In general the systems of interest are nonlinear and, unfortunately, a general global nonlinear theory is not possible because our current understanding of dynamical systems, though extensive, is not adequate for this. However, we can develop a relatively powerful and useful theory based on local analysis about a particular set of parameter values using the extensive and powerful perturbation theory for differential equations. PeTTSy does the most important calculations that underlie such perturbation theory. It provides tools to enable this perturbation theory to be used for the analysis, adjustment, optimisation and design of models including complex models with large numbers of parameters and variables. It allows one to probe the model dynamics and to understand their behaviour under parameter changes. These changes can mimic perturbations to some rates, pulse experiments, or can even mimic the creation of specific mutations such as gene knock-outs or knock-downs.

Moreover, the design of purpose-built add-ons by users or detailed user-designed analysis is enabled by the facility to export all the basic calculation results. For flexibility, results can be exported into the MATLAB workspace, and then further analysis can be done by the user. PeTTSy also provides an interface to XPPAUT [3]. PeTTSy input parameter and initial condition files, or output time series files can be used to generate an input.ode file for XPPAUT. In this way further parameter exploration via simulation within XPP or bifurcation analysis in AUTO can be performed. Moreover, almost all the internal structures of PeTTSy can be exported. This is particularly useful when one is using or designing custom analysis algorithms.

Currently available sensitivity analysis tools [4–7] cater to some of the above needs: however they only deal with a very restrictive set of observables that can be measured (in the case of [4–6]) or only offer insight into systems with steady state dynamics (as in the case of [7]). More importantly, aside from [6] none of the software tools give insight into how temporary changes to parameters can affect the dynamics: hence they cannot describe the effect of pulse experiments or any temporary changes to the systems dynamics. In the case of [6], the output is limited to only changes to the model solution.

PeTTSy has been designed to run simulations and to perform a global form of sensitivity analysis (in the sense of [8, 9]) on the simulated time series. This shows how the model observables (such as the model solution, the period of oscillations, the phase timing or the amplitude) will change as parameters are perturbed either permanently or temporarily.

The methodology we use is system global in that the user can study the impact on the whole time-series (i.e. all model variables simultaneously) or a set of observables of interest rather than being limited to one output at a time.

The versatility of the software is illustrated by the way it has been used in a number of recent papers to engineer systems to have specific complex properties and so aid understanding. For example, it was used in [10] to design a temperature dependent version of the plant circadian clock. It was used to simplify the model so only the most important temperature inputs had to be considered and it was used to understand how the behaviour of the model could be reconciled with the experimentally observed behavior. Another, different application was the use in [11] to understand how to design clocks that are insensitive to external perturbation due to daily fluctuations in light and temperature. In this paper we refer to several of our publications where the software has been essential to give significant biological insight that could later be verified by further experiments.

Another very significant aspect is the ability to implement experimental design or multiple experiments on complex systems via the derivative matrix of the mapping from parameters to the solution of interest and its link to the Fisher Information Matrix. For example, one can use this to design different perturbations of an experiment in order to optimise the amount of information coming from each of these experiments.

We illustrate the use of PeTTSy by analysis of several complex and high dimensional biological models. We will focus on the the clock plant model, counting 28 variables and over a hundred parameters and on the NF- *κ*B model counting 29 parameters and 14 variables. Our aim is to provide an overview of the software, to illustrate its use by considering the analysis of several biological models and to demonstrate PeTTSy’s broad capabilities. Specific technical details of the software are described in the user manual that is available with the software, and the references within.

Toolboxes for sensitivity analysis of ODE models and related areas generally use one of two methodological approaches, deterministic derivative-based methods using mathematical analysis and methods based on sampling of the parameter space. The former is generally considered to be local in parameter space although dynamical systems methods such as bifurcation theory allow one to deduce more global results. Potentially the sampling methods are more global in that they allow exploration of a larger area of parameter space but they are subject to the curse of dimensionality because you need O (*ε*^−*d*^) points in an *ε*-grid to cover the unit disk in *R*^*d*^. An advantage of the derivative-based methods is that they are more directly connected to rigorous results in the mathematical theory, particularly those coming from dynamical systems theory and this is the approach that this paper follows.

Toolboxes employing parameter sampling include SensSB [5], SBToolbox2 (http://www.sbtoolbox2.org/) [12] and DyGloSA [13] and those involving deterministic derivative-based methods include pathPSA [6], AMIGO [14] and Data2Dynamics [15].

Derivative-based toolboxes such as AMIGO and Data2Dynamics analyse systems and fit parameters using a likelihood function that measures the distance between the solution at certain times and corresponding data using a sum of squares of the differences. PeTTSy uses a different approach in that it calculates the linearisation *M* of the mapping from parameters to the solution of interest (i.e. the sensitivity of the model solution to parameters) and then analyses *M* using a number of tools including calculating its principal components and singular values. Though *M* can be calculated in these other toolboxes, most of the PeTTSy analysis depends upon the decomposition of the solution change given in Eq. (1) below and this distinguishes our paper from others. In particular, the sensitivity matrix *S*=(*S*_*ij*_) (defined in Subsection Systems global sensitivity analysis via SVD) is not used in any of those cited above. The detailed justification for using this definition of sensitivity is given in [8, 9]. The graphical plots that then summarise this analysis are specific to this toolbox and include plots for the Singular Spectrum, the Parameter Sensitivity Spectrum, the Sensitivity Heat Map, Time Series Plots with Sensitivity, the Amplitude/Phase Derivatives Scatter Plot and composite plots. Another distinguishing feature from other toolboxes is that the calculation of *M* and the analytical tools mentioned above are developed for periodic orbits.

A key advantage of PeTTSy is that one can export all of PeTTSy’s internal structures for use in the design of purpose-built add-ons by users and for detailed user-designed analysis and design of systems and their properties. For example, PeTTSy routinely calculates the variational matrices *C*(*s,t*) along trajectories between all relevant times *s*<*t* and stores this in a convenient way. Having these matrices at hand enables a large amount of perturbation theory to be practically implemented very efficiently. Finally, we note that PeTTSy fully implements the perturbation theory for periodic orbits.

## Implementation

### Overview

PeTTSy is a MATLAB package, requiring MATLAB R2012a or later to run, the Symbolic Math toolbox, and optionally, the Parallel Computing Toolbox. As such it will run on any platform that MATLAB supports (Windows, Mac OSX and popular Linux distributions). PeTTSy can be freely downloaded from the website: http://go.warwick.ac.uk/systemsbiology/software. Detailed manual documentation is provided with the software. Some of the analysis calculations can be greatly speeded up using MATLAB’s Parallel Computing Toolbox.

The user can also opt to use the CVODES solver [16] (here implemented via sundialsTB MATLAB interface) to speed up the ODE calculations. Solvers can be optimised for stiff or non-stiff problems so the user can determine the best options for their particular model.

**Fig. 1:**
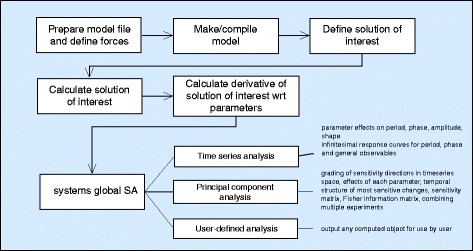
PeTTSy flow chart. This shows the main details of the possible pipelines for the use of PeTTSy

The package consists of command line modules that can be run most easily using the graphical interface that is provided. An overview of the PeTTSy workflows is shown in Fig. 1. The user begins with a model template defining its equations. There are a number of these pre-installed and the manual describes how to create new templates by entering the model equations, or by importing a model from SBML format. The first step is to compile the model using either the make command or the graphical interface. Model derivative matrices are then generated in order to make the model available for analysis. This process employs Matlab’s Symbolic Math Toolbox to create various files that contain symbolic representations of these matrices. Then one defines the solution of interest. For example, for an oscillator this is likely to be an attracting periodic orbit while for a signalling system it may be a solution with a given initial condition.

Note that PeTTSy also allows the export of models to SBML Level 2 format. Model ODEs are converted to SBML rate rules. The required MathML is generated using SnuggleTex, available freely from the School of Physics and Astronomy, University of Edinburgh (http://www2.ph.ed.ac.uk/snuggletex). The required modules are, however, distributed as part of the PeTTSy package so the user need not install SnuggleTex separately.

As well as the differential equation one can define a number of temporal force profiles that can be used to force the system. These are similar to the external factors function of AMIGO and the experimental treatment function of Data2Dynamics. A typical use of these would be when modelling a forced oscillator such as a circadian oscillator entrained by light or temperature, but they can also be used to model experiments where the system is perturbed artificially such as when external signals are used to synchronise a system or induce expression of specific genes.

The software then finds this solution of interest. The second step is to accurately compute it. For example, if this is a periodic solution the software first runs the model with the specified parameters and initial conditions to find it approximately and then uses a boundary value solver to increase the precision.

The third step is to solve the variational equation along this solution and to use this to calculate the derivatives of the solution of interest with respect to all the parameters. These steps are needed for all the subsequent analysis. At this stage one already has several classical (e.g. period, phase and amplitude derivatives) and new (e.g. infinitesimal response curves (IRCs) and phase IRCs) analysis tools available. These outputs can be selected using the graphical interface. This process involves significant computation and therefore using the graphical interface one can configure what aspects need to be computed and see how accurate the computation is likely to be. For the latter, after being presented with an informative analysis, the user can opt to re-calculate the fundamental matrices (the building blocks of the analysis, described in more detail in the manual) by increasing the time resolution at which the computations are done. More details about the time resolution required are outlined in the accompanying manual. If the user has a copy of the MATLAB Parallel Computing Toolbox installed, then they will be given the option of running the analysis using one of the user defined parallel configurations. The computation of the fundamental matrices is ideal for parallelisation and a substantial speed-up can be achieved.

The fourth step is to use the global sensitivity analysis tools as described below. This gives a systems global picture of all changes that occur as parameters are changed and presents this in a way that grades the effects so that one can gain better understanding. This uses a version of Singular Value Decomposition (SVD). An optional fifth step is to use the software for experimental optimisation.

**Fig. 2:**
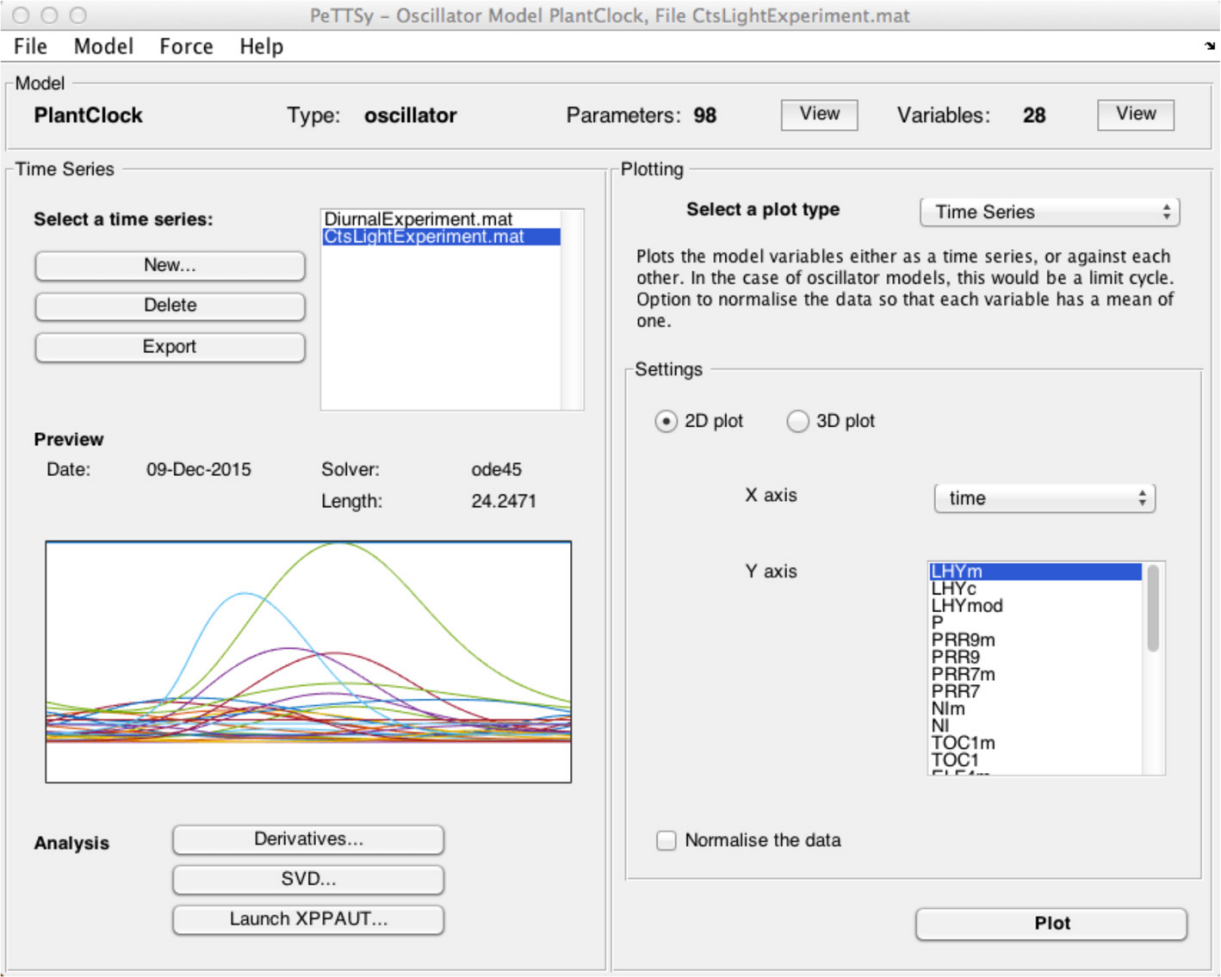
First GUI (’PeTTSy’). The graphic user interface provides an easy way for the user to run the simulation and perform sensitivity analysis for the models. Once the user has run at least one simulation, the results are saved and the user can access the time series (in this case labeled “DiurnalExperiment” for 12 hour light and 12 hour dark series and “CtsLightExperiment” for a model with lights permanently on). Plot of the time series is provided below, and the user can also plot variables separately, or as a 3D plot (by specifying the desired plot specifications in the Plotting subsection)

All of these operations can be carried out via command line operation or via the extensive graphical interface (Fig. 2). This enables the user to define and manipulate all models choices, inputs (e.g. parameter values and initial conditions), definitions and computations and present the outputs graphically or numerically. PeTTSy allows the automated plotting of the results of all analyses, including the ability to select the parameters and variables of interest. Results are saved to file in MATLAB format, but in addition can be exported to the MATLAB workspace. This allows the user to save them in any format they wish for post-processing.

### Time series analysis

After calculating the solution derivatives the user can choose to display the following plots:

#### Solution derivatives

Suppose we are studying an ODE model

$$\frac{dx}{dt}=f(t,x,k). $$

Here *t* is time, the vector *x*=(*x*_1_,…,*x*_*n*_) represents the model variables and the vector *k*=(*k*_1_,…,*k*_*s*_) represents the model parameters. We denote by *x*=*g*(*t,k*) the solution of interest which is taken to be determined over a specific time range 0≤*t*≤*T*. For oscillator models, the time *T* will be the period of the limit cycle oscillation, while for signalling systems *T* will be the length of the time the model is simulated. For the *i*-th variable of the solution *g*(*t,k*) the solution derivative with respect to the *j*th parameter evaluated at time *t* is (*∂g*_*i*_/*∂k*_*j*_)(*t*).

Given a differential equation of the above form there is a unique solution *ξ*(*t,t*_0_,*x*_0_,*k*) defined by the initial condition *ξ*(*t*_0_,*t*_0_,*x*_0_,*k*)=*x*_0_. In signalling systems such as the oft-studied NF- *κ*B system, the solution of interest is a solution of the form *ξ*(*t,t*_0_,*x*_0_,*k*) where *k* is the parameter vector relevant to the stimulated situation and *x*_0_ is the equilibrium solution found when the parameters take the value relevant to unstimulated conditions. Thus the solution of interest is defined by the fixed initial condition. If, on the other hand, we are interested in a periodic solution *g*(*t,k*) then *g*(*t,k*)=*ξ*(*t,t*_0_,*g*(*t*_0_,*k*),*k*). Thus, the initial condition depends upon the parameters *k* and is implicitly defined.

For signal models, the derivatives will always be non-periodic, and for forced oscillators they will always be periodic. For unforced oscillators, the derivatives will generally be non-periodic because the period changes with parameters. However, if instead of considering *g*(*t,k*), one replaces it with $\bar {g}(t,k)=g(\lambda t,k)$ with *λ*=*λ*(*k*) chosen so that the period is locally independent of parameters, then the derivative $ ({\partial \bar {g}_{i}}/{\partial k_{j}})(t) $ is periodic and tells one how the shape of *g*(*t,k*) changes with parameters. The user can plot non-periodic or periodic derivatives. Further details of the above scaling are outlined in the manual. Details about the theory behind all this are given in [9].

#### Period derivatives

This plot is only relevant for unforced oscillators. In the software this derivative is obtained from the IRC curves described below in section Infinitesimal response curves (IRCs) integrated over the full time interval i.e. *ϕ*_1_=0 and *ϕ*_2_=*τ* (with the full description given in the SI). Theory behind this calculation is explained in [17].

The user can select different scaling of the period derivative, for example looking at relative change rather than absolute change, as well as which values to plot, and these can be sorted by value or parameter name.

#### Phase derivatives

This plot type applies to entrained forced oscillators only. For such systems the period is invariant provided the system stays entrained but the phase of the periodic solutions can change as parameters are varied. The phases *ϕ* measured are the times of the peak and troughs. The phase derivatives with respect to model parameters (i.e. *∂ϕ*/*∂k*_*j*_) are plotted as a bar chart. There are options to plot derivatives with respect to log parameter, and to represent the resulting derivative on a logarithmic scale and there are several other plotting options. A description of the derivatives is given in the SI.

#### Infinitesimal response curves (IRCs)

This plot type applies only to unforced oscillators. It plots the period change produced by an infinitesimally small perturbation of the parameter at a single time point during the free running limit cycle. More specifically, for a oscillator with a stable limit cycle with period *τ*, changes to some output of interest *Q* (such as period or phase *ϕ*), given by *δQ*, can be described as a linear combination of changes to each parameter *k*_*i*_ (described by *δk*_*i*_) of the form,

$$\delta Q= \sum_{i=1}^{s} \delta k_{i} \left(\int_{\phi_{1}}^{\phi_{2}} f_{k_{i}, Q_{j}} (\phi) d\phi \right). $$

Here the function $ f_{k_{i}, Q_{j}} $ is the infinitesimal response curve (IRC) for parameter *k*_*i*_ on the output *Q*_*j*_, and the changes to the parameters have only between applied over the time window *t*∈[*ϕ*_1_,*ϕ*_2_]. Namely, *k* is changed to *k*+*δk* where *δk*=(*δk*_1_,…,*δk*_*s*_) only during the time [ *ϕ*_1_,*ϕ*_2_]. For more details the user is referred to [8, 17]. A different but related IRC is also calculated by the toolbox pathPSA [6].

The user can sort parameters by maximum phase advance, maximum phase delay or by the areas under the IRC curve. The integral of the IRC is equal to the derivative of the period of the limit cycle with respect to that parameter. A value of zero indicates that a permanent change to that parameter would cause no overall change to the phase or period of the limit cycle. A positive value means an overall phase advance (and period shortening), and a negative value an overall phase delay (and period lengthening). IRCS can be plotted as heat maps or as line plots.

A practical example of the use of these IRCs is given in [8, 17] where they are used to study temperature compensation and show that the imposition of temperature compensation (invariance of period under sustained temperature changes) does not conflict with the need for entrainment by daily temperature oscillations (susceptibility of a clock to such variations).

#### Phase infinitesimal response curves (phase IRCs)

This plot type applies only to forced oscillators. The phase infinitesimal response curves represent changes to phases (usually defined as peak or trough times) of the model variables in response to parameter perturbations. Note that period of forced oscillators is fixed by the external force when they are entrained and so it will remain constant under parameter perturbations. Each phase IRC has a discontinuity at the time of the peak in question. Change of phase is represented by summing the integral of the phase IRC and a partial derivative, for details refer to the Appendix, that is represented on the plots as a single point drawn at time of phase. The rest of the interpretation of how phase changes as parameter is perturbed follows similar lines to the interpretation of the IRCs. Namely, if the selected parameter is perturbed over the whole limit cycle then change in phase is indicated by the area under the whole phase IRC and the single time point value. If the parameter is perturbed over the time interval that does not include the time of the phase, then the phase change is given only by the corresponding area under the phase IRCs curve. For a permanent perturbation of the parameter, if the area under the phase IRC is positive and the aforementioned single point value is positive, then there will be a phase advance. Likewise, if both are negative, this will result in a phase delay. However, if they are found to be of opposite sign, then determining whether the perturbation will result in a phase advance or delay requires examination of their actual values. Phase IRCs apply to a particular variable, and so the user must specify the variable of interest, and in the case of multiphasic variables, specify the peak of interest too. As for the IRCs, the user can sort parameters by maximum phase advance or maximum phase delay in response to a parameter change at a single time point. However, rather than area under the IRC curve, instead total phase change values are displayed for when the parameter is perturbed permanently, i.e for the whole of the limit cycle.

### Systems global sensitivity analysis via SVD

After calculating the solution derivatives, SVD analysis can then be performed in order to investigate global sensitivity. This is done in order to analyse the linearisation of the mapping from parameter perturbations *δk*∈*R*^*s*^ to changes *δg* in the solution of interest. This is a map into an infinite dimensional space of smooth functions. PeTTSy approximates these functions by the high-dimensional vectors given by evaluating the function on a very fine grid of times. This is a good approximation as the functions are generally very smooth and the grid is chosen appropriately. If there are *N* of these times, then *M* is represented by a *nN*×*s* matrix (*n* state space dimension and *s* the number of parameters). We denote this matrix by *M*=*∂g*/*∂k*. To understand why analysis of this is likely to be useful for experimental optimisation note that *F*=*M*^∗^*M* is the Fisher Information Matrix for a natural stochastic extension of the model being considered.

SVD decomposes *M* as

$$M = U\Sigma V^{T} $$

where *U* is an orthogonal matrix whose columns are the principal components (PCs), the matrix *σ* is diagonal with the singular values, *σ*_*i*_, along the diagonal, and the columns *V*_*j*_ of the matrix *V* form an orthonormal basis for parameter space that provides information for the construction of the Sensitivity Heat Maps (SHMs), detailed later on. They are the eigendirections for the Fisher Information Matrix *F* introduced above.

The *principal global sensitivities* are the numbers *S*_*ij*_=*σ*_*i*_*W*_*ij*_ where *W* is the inverse *V*^−1^=*V*^*t*^ of *V*. Note that $\sum _{j} S_{ij}^{2}={\sigma _{i}^{2}} \sum _{j} W_{ij}^{2}={\sigma _{i}^{2}} $. In [8, 9] it is shown that for the change *δg* of the solution *g*(*t,x*) due to a change *δk*=(*δk*_1_,…,*δk*_*s*_) in the parameters, can be written as

(1) $$\begin{array}{@{}rcl@{}} \delta g &=& \sum_{i,j} W_{ij}\delta k_{j} \sigma_{i} U_{i} +O\left(\left\| \delta k^{2}\right\|\right) \end{array} $$

(2) $$\begin{array}{@{}rcl@{}} &=& \sum_{i,j} S_{ij}\delta k_{j} U_{i} +O\left(\left\| \delta k^{2}\right\|\right) \end{array} $$

where *σ*_1_≥*σ*_2_≥…≥*σ*_*s*_≥0 are the singular values, the *U*_*i*_ are the principal components and the *W*_*ij*_ are the entries of *W*. A key relation that follows from this is

$$\| \delta g \|^{2} = \| S\cdot\delta k\|^{2}, $$

see [9]. Note also that the above mentioned Fisher Information Matrix equals *S*^*t*^*S*.

The analysis workflow in this section of PeTTSy uses the basic module of an *Experiment*. Let us try and make this notion clear and explain why we use this terminology. For a given system such as the circadian clock we may have a single model but in related experiments we may modify this in various ways. For example, we may knock out genes, alter the forcing by light or temperature, and biochemically alter rate constants. To model each case we would modify the model somewhat. For example, to model a gene knockout we might set the transcription or translation rate of that gene to zero. To work out the value that one of these experiments adds to the others we would need to construct a big model that combines all the individual ones. PeTTSy effectively does this. Although a single experiment is a single parameterised model in the usual sense, as explained below, when they are combined one can ask interesting questions. PeTTSy calculates all of the above quantities for the combined system and therefore enables a quantitation of the value mentioned above. An advantage of the way that PeTTSy does this comes from the fact that to add an extra experiment to a set of them that have been previously calculated, one only has to calculate the derivative matrix for the new experiment and then concatenate it to the previously calculated matrices and then calculate the SVD of the combined matrix (a fast operation).

This approach which is implemented in PeTSSy could be used in other toolboxes such as AMIGO and Data2Dynamics that implement the calculation of the linearisation matrix *M*.

**Fig. 3:**
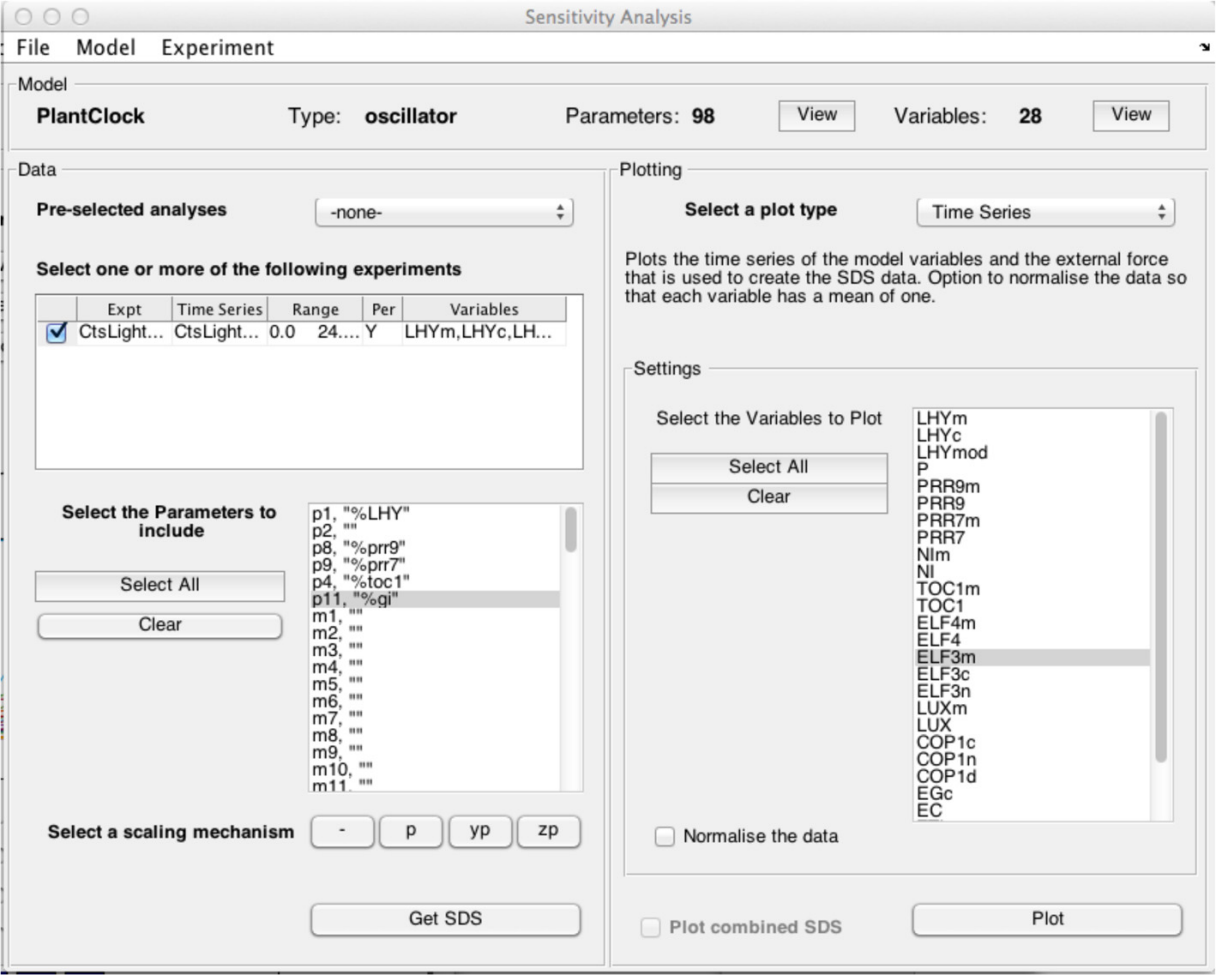
Second GUI (’Sensitivity Analysis’). The graphic user interface provides an easy way for the user to select an ’Experiment’. In this case, the ’Experiment’ generated is called ’CtsLightExpt’. The user can select which parameters to include in the SVD analysis and once SVD is done, can select a plot type (details of the plot types and plot specifications are outlined here as well as in the manual)

There are many output options for this analysis, so it has its own GUI, see Fig. 3. As in the main PeTTSy GUI, the desired model can be selected via the menu system. Analysis is then performed on the experiments both separately and combined.

There are a number of variations included in the package that allow one to focus on different aspects. Firstly, because in many models the actual parameter values *k*_*j*_ can differ by orders of magnitude it includes an option where all calculations use the logged parameters so that one is studying relative rather than absolute changes. It also offers an option of using the relative change for the solutions of the system. In addition, for solutions of unforced oscillators one can opt to decompose the change in the solution into a change in period and a change in shape of the solution. One can also choose selected time points to include, which can be done graphically by clicking on a time series plot. Then one is just looking at the variation of *g* at these timepoints. This enables one to optimise the choice of timepoints when doing experimental optimisation. Similarly, one might want to only include certain variables or form composite variables by forming linear combinations of two or more. This allows the user to replicate experimental conditions. For example, a model may include a particular protein in both the nucleus and cytoplasm but the experimental data may only include the overall levels in the cell and so the user would then want to sum the two model variables. All of these aspects are built into PeTTSy.

#### Classical sensitivity coefficients

Classical sensitivity coefficients ${C^{Q}_{j}}$ for some output of interest *Q* (e.g. period or amplitude of unforced oscillator) can be written in terms of *S*_*ij*_ and *U*_*i*_ components because

$$C^{Q}_{j}\cdot \delta k = L\cdot \delta g $$

where *L* is the linear operator that associates to a change *δg* in the solution the corresponding linearised change in *Q*. This is calculated for all the obvious *Q* within PeTTSy. A detailed derivation and list of all of them is provided in [8].

#### Experimental optimisation

Before performing the SVD analysis the user chooses which experiments will be included, which variables or combintions of variables and which time points. These actions will change the matrix *M* that will be analysed, namely it uses row operations on the matrix (in the case of creating new variables) or removal of relevant rows of the *M* matrix (when one wants to remove eliminate some variables from the experiment because, e.g. they cannot be measured).

In particular, one can compute the Fisher Information Matrix *F* for each combination of the various experiments. Usually, one is interested in seeing the extent to which a new experiment or set of experiments increases the eigenvalues of *F* or decreases their decay. These are given by ${\sigma _{i}^{2}}$ where the *σ*_*i*_ are the singular values introduced above. PeTTSy displays the relevant information to enable this.

#### Displaying the outputs

The Plotting panel allows the user to select a variety of ways to view the input to and output from the SVD analysis. The first two, Time Series and Solution Derivatives, are similar to the corresponding plots in the main PeTTSy GUI, except that they relate to the selected experiment(s) rather than the raw time series file, and so show the effect of combining and omitting variables and selecting time points.

The other main graphical outputs from the global analysis include the following:

##### Singular spectrum plot.

This plots the largest singular values so that the user can assess how fast they decay. The user can choose how many are plotted.

##### Parameter sensitivity spectrum (PSS).

The PSS plots the matrix of the principal global sensitivities *S*_*ij*_=*σ*_*i*_*W*_*ij*_. This spectrum can be plotted as either a 3-dimensional surface plot or bar chart (parameter, *k*_*j*_, vs PC index *i*, vs *S*_*ij*_, or as a series of 2-dimensional charts (plotting parameter against the *S*_*ij*_) one for each PC. The user is able to sort by parameter and select the most important to plot, and also to choose whether to plot the raw spectrum, absolute values or log10 absolute values. When performing experimental optimisation, a separate plot is produced for each experiment, plus an additional plot for the combined matrix. One is able to view how combining experiments changes the spectrum. This is a particularly useful plot as from it one can immediately see which are the most sensitive parameters and how they affect the global solution.

##### Sensitivity heat map.

Sensitivity heat maps (SHMs) are used to identify what variables *g*_*m*_ are most sensitive to parameter variation and the temporal profile of how this sensitivity manifests itself. This information can be used to determine which outputs *Q* have high coefficients and for which parameters, *k*_*j*_. Instead of inspecting the variation in the solution one can also do the same for the principal components *U*_*i*_. In the former case one plots

(3) $$\begin{array}{@{}rcl@{}} f_{i,m}=\left(\max_{j} |S_{ij}|\right) |U_{i,m}(t)| \end{array} $$

and

(4) $$\begin{array}{@{}rcl@{}} f^{d}_{i,m}= \left(\max_{j} |S_{ij}|\right) |\dot{U}_{i,m}(t)|. \end{array} $$

We choose these because the sensitivities ${C^{Q}_{j}}$ discussed above can be written as linear combinations of terms of the form *S*_*ij*_*U*_*i,m*_(*t*) and $S_{ij} \dot {U}_{i,m}(t)$ for all the usual choices of the observable *Q*. For the latter case instead one plots the variables from the scaled principal components, *σ*_*i*_|*U*_*i,m*_(*t*)|.

These time series *f*_*i,m*_ and *σ*_*i*_|*U*_*i,m*_(*t*)| are plotted as a heat map with colour representing value and distance along the heat map representing time. There is an option to select the most important variables, defined as those with a maximum sensitivity value (*σ*_*i*_|*U*_*i,m*_(*t*)|,*f*_*i,m*_ or $f^{d}_{i,m}$) exceeding a specified proportion of the global maximum. It is also possible to mark the most important regions on each heat map, again defined as those exceeding a specified proportion of the global maximum.

When performing experimental optimisation, two figures are produced for each experiment, one representing the experiment analysed on its own, and one representing this experiment’s component of the combined matrix.

##### Time series with sensitivity plot.

This plots the selected time series showing which parts are sensitive and to what parameters.

##### Composite plot.

The composite plot combines several of the above plot types. These are: the sensitivity values (*σ*_*i*_*U*_*i*_(*t*) or *f*_*i,m*_(*t*)) for the selected variable and PC, plotted as a heat map and line plot; derivatives ($\sigma _{i} \dot {U}_{i}(t)$ or $f^{d}_{i,m}(t)$, respectively) plotted as a heat map; time series of the selected variable; and the PSS for the chosen principal component, *σ*_*i*_*W*_*ij*_ (for all *k*_*j*_). This allows the user to view the sensitivities in a compact form. When performing experimental optimisation two figures are produced, one representing the selected variable/PC combination for the experiment that the variable was taken from, and a second plot representing this experiment’s component of the combined matrix.

##### Amplitude/phase derivatives scatter plot.

This plot visualises the extend to which the change produced by a parameter variation is a simple phase change or an amplitude change. Effectively it takes the change *δg*_*m*_ in the *m*th variable and decomposes it as *α*_*m*_*R*_*m*_+*β*_*m*_*A*_*m*_+*S*_*m*_ where *R*_*m*_ is the unit vector that represents an infinitesimal translation by time, *A*_*m*_ is an infinitesimal amplitude change of *g*_*m*_ and *S*_*m*_ is a vector orthogonal to *R*_*m*_ and *A*_*m*_. It then plots the pair (*α*_*m*_,*β*_*m*_) for any user-defined subset of the variable indices *m*. A detailed derivation of *R*_*m*_ and *A*_*m*_ is given in the Additional file 1. Instead of doing this for the variable changes *δg*_*m*_ one can do it for the principal component *U*_*i*_ and thus determine to what extent they are a simple translation or an amplitude change.

## Results and discussion

### Model time series and solution derivatives

PeTTSy has been applied to a broad range of examples (with specific details further on in the relevant sections), but for purposes of illustrating the software we will be applying it to two exemplar systems: the plant circadian clock model of [18] and the model of NF- *κ*B oscillations from [19].

**Fig. 4:**
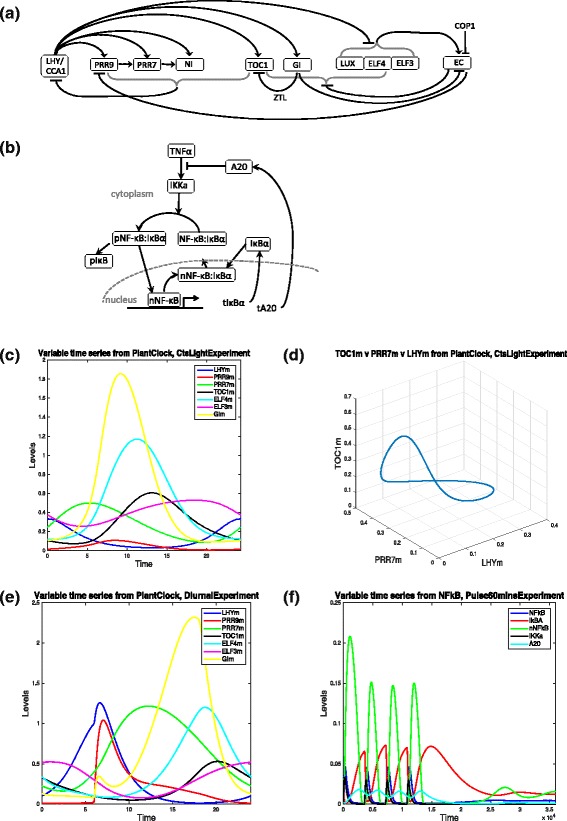
Plant clock [18] and NF- *κ*B [19] network diagrams and different plot options for their model times series. **a** Plant circadian clock network of [18]. **b** NF- *κ*B signalling network from [19]. **c-d** Simulated time series of different models in PeTTSy. Several mRNA time series of the plant circadian clock under constant light in (**c**) 2D and (**d**) 3D. **e** Several mRNA time series of the plant circadian clock under 12 h light and 12 h dark cycles (photoperiodic forcing). **f** Several time series of NF- *κ*B signalling system [19] under four 5 min pulses administered every 60 min and then no pulse for the remaining 400 min (24000 sec)

The plant clock model consists of 28 variables representing the mRNA and protein levels of the genes LHY, CCA1, TOC1, PRR9, PRR7, NI, LUX and ELF4; ZTL protein, LHY modified protein; mRNA of ELF3 and GI, cytoplasmic proteins of ELF3, GI, COP1; nuclear proteins of ELF3; GI and COP1 in day and night forms; and the cytoplasmic protein complexes ELF3-GI, GI-ZTL (ZG) and nuclear protein complexes ElF3-GI, ELF3-ELF4, and EC. The model has a complex structure that consists of multiple positive and negative feedback loops and contains 104 parameters (see Fig. 4(a)). Note that 6 of the parameters are Hill function coefficients so we will keep them at a fixed value (given in [18]) and we will not vary them, thus reducing the parameter dimension to 98. We will simulate the model subject to two different experimental perturbations: constant light (Fig. 4(c) and (d)) and 12 h light and 12 dark conditions (Fig. 4(e)).

For an exemplar signalling system we use the NF- *κ*B model of [19]. It describes the oscillations in the level of cytoplasmic and nuclear NF- *κ*B concentration resulting from an incoming signal of tumor-necrosis factor- *α*, TNF- *α*. We consider both the effect of constant stimulation by TNF- *α* and of pulsatile TNF- *α* stimulation using a 5 min pulse every 60 min. The model has 14 variables describing cytoplasmic and nuclear NF- *κ*B, I *κBα*, their complexes, A20 mRNA and protein and the kinase IKK in its activated and inactivated states. The IKK system is activated by TNF- *α* and goes on to cause phosphorylation and subsequent degradation of I *κBα* freeing NF- *κ*B to move into the nucleus. In the nucleus NF- *κ*B activates I *κBα* transcription subsequently producing I *κBα* protein that bind the nuclear NF- *κ*B and exports it back into the cytoplasm, causing the process to repeat (Fig. 4(b)).

Figure 4(c-f) illustrates the different plotting options discussed in Section Time series analysis applied to the two models and the time series generated for both.

**Fig. 5:**
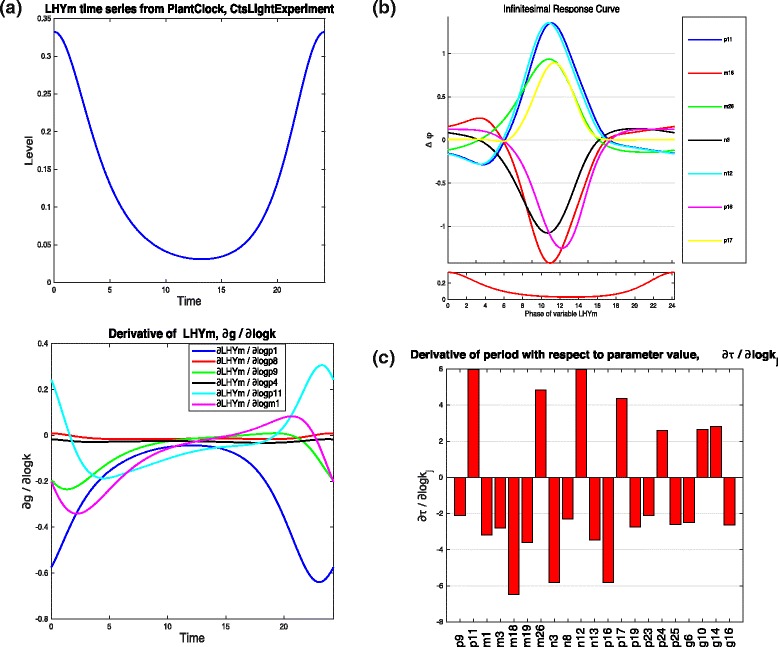
Time series, IRC and Period derivative plots. **a** Top panel, the time series for LHY mRNA from the plant circadian clock model plotted for one cycle of the oscillation. Bottom panel, the solution derivatives of LHY mRNA with respect to different parameters. **b** Infinitesimal response curves for top seven parameters and an inset (below) of the time series of the first variable of the model, LHY mRNA. **c** Period derivatives for 21 parameters with the largest period derivative values

For both models we are interested in changes to the solutions that are brought about by changes to model parameters. Suppose that we are interested in understanding the behaviour of LHY mRNA levels of the plant clock model (Fig. 5(a)) under various parameter perturbations. In Fig. 5(a) (lower panel) we show the periodic solution derivatives of LHY mRNA with respect to six parameters: *p*_1_, *p*_8_, *p*_9_, *p*_4_, *p*_11_ describing the translation of LHY, PRR9, PRR7, TOC1 and GI protein, respectively, and *m*_1_, the degradation rate of LHY mRNA.

The results show that increasing the LHY mRNA degradation (*m*_1_) will lower the peak levels of mRNA (around times close to *t*=0 and *t*=24). Increasing the translation of repressors of LHY mRNA, namely TOC1 and LHY protein, represented respectively by parameters *p*_4_ and *p*_1_, also lowers the amplitude of LHY mRNA oscillations (note that period solution derivatives are all negative close to *t*=0 and *t*=24).

On the other hand, small increases in translation of repressor PRR9 (parameter *p*_8_) has almost no effect on the LHY, while increasing the translation of GI protein (parameter *p*_11_) will counteract the effect of the repressor PRR7 (parameter *p*_9_) and raise the level of LHY mRNA amplitude. Extracting this information from the network scheme itself is difficult: GI plays a dual role of an implicit activator of LHY, via TOC1, and its repressor, via EC and PRR9 (refer back to Fig. 4). Our results show that in this case GI will acts as an overall activator, and this could be down to the fact that activation goes via fewer intermediaries. Furthermore, though PRR9 and PRR7 are repressors, changes to their translation rates appear to be less striking than changes to translation of TOC1 and GI repressors of LHY. This insight cannot be gained from the network diagram alone.

The real power of this approach is that in fact, in order to explore the effects of a simultaneous change to several parameters, one just has to combine the effects of each solution derivative for each parameter of interest [17]. The combination is just a linear sum of the solution derivatives where the weights of the sum describe the desired percentage change of each parameter. So, in Fig. 5(a), any increases in the translation of GI protein (parameter *p*_11_) will counteract any effect of increases to the other five parameters. Whether the actual LHY mRNA peak increases or decreases will be subject to how much each parameter is changed.

The solution derivatives can be used as a predictive tool to show how combined parameter changes will affect the model dynamics (without performing tedious manual changes by hand first) and what sort of experimental data features the model will be able to match under these parameter changes. For a further example of this type predictive analysis, we point the reader to our paper [10] (c.f. Table 2 and Additional file 1).

### Response curves and derivatives for the plant circadian clock

Oscillatory behaviour and any changes to this behaviour are of interest when probing the plant circadian clock. The infinitesimal response curves (IRCs) are a useful tool from which two types of information can be extracted: (i) the effect of a permanent or temporary change to a parameter value on the period of oscillations, *τ*, and (ii) the effect of such a change to a parameter value on the phase advance or delay of oscillations.

Figure 5(b) top panel shows the IRCs for the following parameters of the plant clock model: namely, GI transcription and translation (*n*_12_, *p*_11_), ELF3 mRNA degradation (*m*_26_) and formation of ELF3-GI complex (*p*_17_), GI mRNA degradation (*m*_18_) and ELF3 transcription and translation of cytoplasmic protein (*n*_3_ and *p*_16_, respectively). Period derivative *∂τ*/*∂k*_*j*_ takes the value of the signed area under the IRC curve for parameter *k*_*j*_ [8]. From Fig. 5(b) the signed area under the IRC for parameter *p*_11_, representing the translation of GI, is positive, hence a permanent increase of this parameter will lead to an increase in the period of oscillations, as observed in Fig. 5(c). On the other hand, a permanent increase in GI degradation will have the opposite effect on the period.

From Fig. 5(b), the IRCs for all the parameters shown reach their maxima or minima around the time of the trough of LHY mRNA levels (shown in lower panel on Fig. 5(b) and likewise are close to zero around the times when LHY mRNA levels are high (i.e. around phase *ϕ*=0 and *ϕ*=24). This means that an introduction of an infinitesimal (short term) perturbation of parameters when LHY mRNA levels are low will elicit a greater phase shift of the oscillations than a perturbation introduced when levels of LHY mRNA are high. This information can be used to check the model response to pulse experiments and to create phase response curves (the reader is referred to [17] (c.f. Figure 2) where the authors show how approximations of the PRC obtained from the IRC closely match the measured PRCs of a Drosophila circadian clock model of [20]). In general terms, a change to phase can be obtained by taking the negative of the value of the signed area under the relevant IRC for the relevant length of the perturbation. In Fig. 5(b), a short increase of GI translation for a short period administered approximately 10 hours after the peak of LHY mRNA will elicit a phase advance of the clock, while a short increase in GI mRNA degradation will have the opposite effect.

Period derivatives summarise the information provided by IRCs on the question of the effect of a permanent change to a parameter value on the period of oscillations, *τ*. As explained above, IRCs are used to calculate the period derivatives. The plot of period derivatives for the plant clock model for the top 20-odd parameters with greatest effect on period is shown in Fig. 5(c). The period is most sensitive to dynamics of genes GI and ELF3, with parameters having greatest effect being those related to GI transcription and translation (*n*_12_, *p*_11_), ELF3 mRNA degradation (*m*_26_) and formation of the ELF3-GI complex (*p*_17_), as well as GI mRNA degradation (*m*_18_), ELF3 transcription and translation of cytoplasmic protein (*n*_3_ and *p*_16_, respectively). While increasing the value of the first four aforementioned parameters will increase the period, doing the same to the last three will decrease the period of oscillations. This is comparable to the information provided by the IRCs.

For the plant clock models, period information is very important, since for the clocks, the ability to maintain near-constant period across changing temperatures is a key feature of the system. In circadian literature this feature is referred to as temperature compensation. Models of temperature-compensated clocks can be designed by ensuring that the model parameters change according to specific balance equations of [21] that rely on the information from period derivatives. In a recent paper [10] the authors used period derivatives calculated by PeTTSy to construct and parametrize a model of a plant clock that can temperature compensate. Further information on this can be found in [10].

**Fig. 6:**
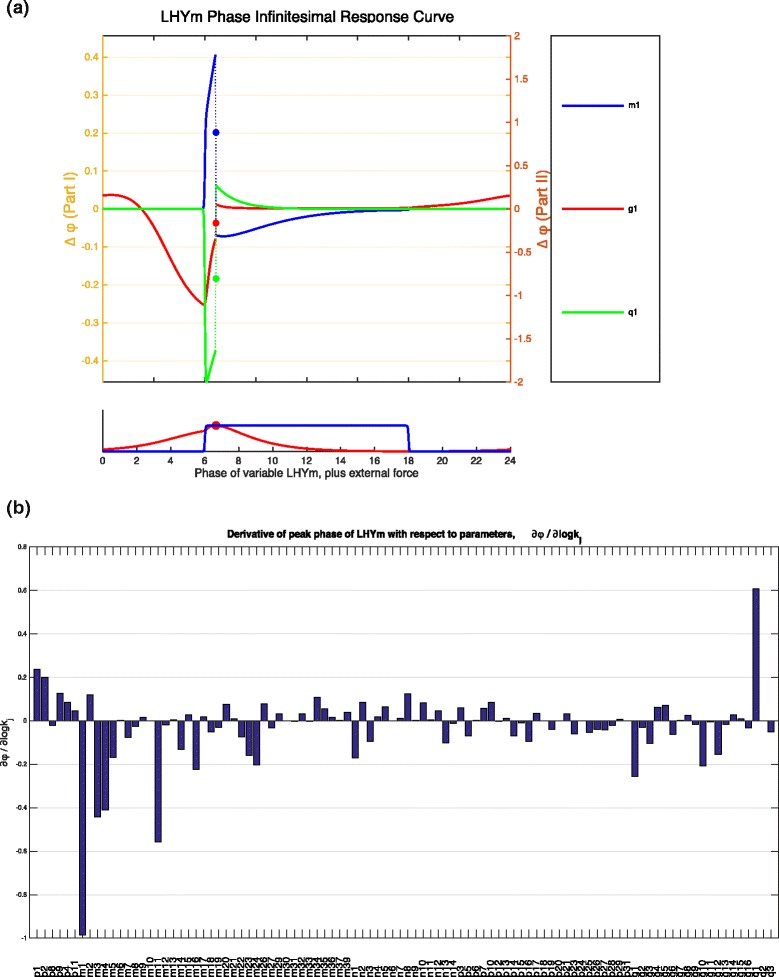
Phase IRC and phase derivative plots. **a** Phase IRCs for three parameters of the plant clock model under diurnal forcing with 12 h light and 12 h dark periods with an inset of the time series of the variable of interest, LHY mRNA. **b** Phase derivatives of the LHY mRNA with respect to all model parameters

In case of plant clocks subject to entrainment by some sort of forcing, such as day-night cycles of light or temperature cycles, the interest is not in the period of oscillations (since these are predetermined by the force applied) but in the changes to phase (i.e. the time of maximum of minimum of expression levels) that can occur. In Fig. 6(a) we show the phase IRCs for LHY mRNA of the plant clock model subject to 12 hour light: 12 hour dark cycles. We plot the phase IRCs for several parameters whose permanent perturbation was identified in the last subsection as having the greatest effect on the period of LHY mRNA, Fig. 5. Note that a permanent perturbation to parameters for LHY mRNA light-degradation and transcription (*m*_1_ and *g*_1_, respectively) has a similar effect on LHY mRNA phase, but any shorter temporal perturbation of each one will elicit a different phase change in LHY, Fig. 6(a). We can plot the zoomed figure of each phase IRC separately (not shown). An infinitesimally perturbation of *m*_1_, administered after the peak of LHY mRNA and during the time that the lights are on (from time 6 h up to 18 h) will lead to an phase advance (negative *Δϕ*) of LHY mRNA. A slightly longer duration pulse, say starting 4 h after lights are on (i.e. at time 10 h) will also lead to a phase advance. On the other hand, a duration of perturbation to *g*_1_ with the same timing will result instead in a very small phase delay (though this delay is so small that it is close to zero). This example shows us that though a permanent perturbation of each parameter will have the same effect, the shorter perturbations of each parameter over specific times in the oscillation cycle can in fact lead to a very different response.

As in the case of period IRCs and period derivatives, the phase IRCs information about the effect of a permanent parameter change on model phase, is summarised in the phase derivatives. We show the plot of phase derivatives for the plant clock model subject to LD forcing in Fig. 6(b) for all the model parameters. Here the phase derivative graph shows that the phase of LHY mRNA is most sensitive to rates describing LHY transcription and mRNA light-dependent degradation, protein degradation and translation (*g*_1_, *q*_1_, *m*_1_, *m*_3_ and *m*_4_, *p*_1_), as well as degradation of light-active protein P (*m*_11_). It is not surprising that parameters describing LHY dynamics feature at the top of the list. Other parameters that the model is sensitive to are related to dynamics of NI protein, a repressor of LHY (parameters *m*_16_ and *m*_24_ describing NI mRNA and dark-dependent protein degradation) and LHY dark-dependent translation (*p*_2_). What is surprising is that only a dozen or so parameters have any significant effect on the phase of LHY mRNA peaks, with only a handful have any significant effect. As would be expected, increasing the rate of mRNA light-degradation (*m*_1_) leads to an earlier peak in LHY mRNA (seen as a negative phase derivative). Effect of changes to LHY protein dynamics on the mRNA is harder to interpret using the network structure alone, since LHY has a negative feedback on itself via other morning loop genes (the PRRs) and many of the evening loop genes, but appears to have an overall positive feedback via evening gene TOC1 (by repressing the TOC1 repressor). Information from the phase derivatives indicates that increase in translation of LHY (*p*_1_, *p*_2_) will delay the peak of LHY mRNA, while increasing the rates of the LHY protein and LHY modified protein degradation (*m*_3_, *m*_1_ and *m*_4_) will advance the phase.

### SVD analysis of the plant clock model

**Fig. 7:**
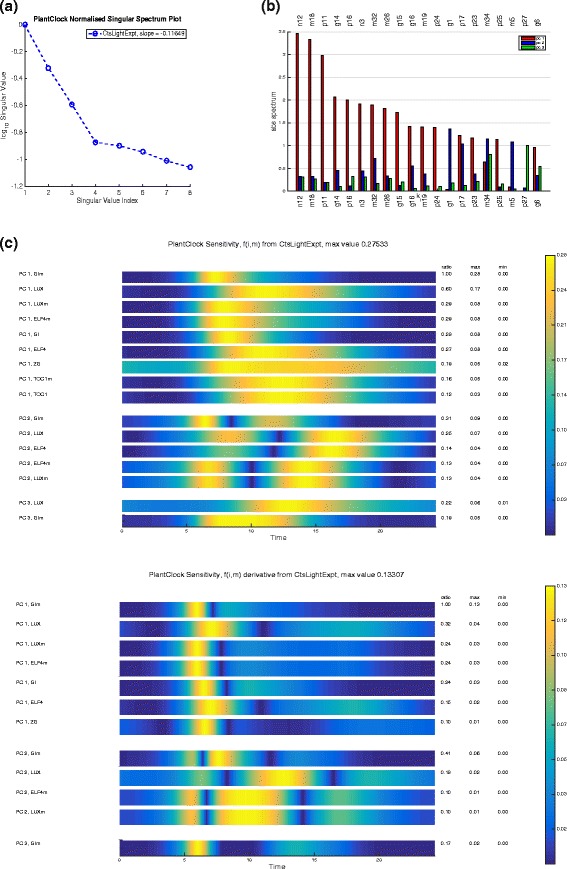
Singular value spectrum plot, the PSS and the SHMs. **a** Singular value spectrum plot of the top 8 singular values ranked in order of decreasing value. **b** The PSS with each group of bars corresponding to the value of |*σ*_*i*_ *W*_*ij*_| for any parameter *k*_*j*_. Only those for which the values are significant are plotted, i.e. for *i*=1,2,3. The parameters *k*_*j*_ are ordered by max*i*=1,2,3|*σ*_*i*_ *W*_*ij*_| and only top 20 are plotted. **c** Sensitivity heat map showing *f*_*i,m*_ (top panel) and $f^{d}_{i,m}$ (bottom panel). The threshold is set to be 10 % of the global maximum of *f*_*i,m*_(*t*). The only values of principal components (indexed by *i*) and variables (indexed by *m*) for which max*t* *f*_*i,m*_(*t*) is greater than this threshold have *i*<=3. These are ranked in order of decreasing size with the ratio change also given. The plotted *f*_*i,m*_ and $f^{d}_{i,m}$ are coloured on the scale where their amplitudes are scaled to 1

We start by getting an overview of the sensitivity of the plant clock and initially study the clock in constant light. Plotting the log10 singular values with them normalised by the maximum singular value shows that they decrease rapidly (Fig. 7(a)). Already the third singular value is a bit more than only 20 % of the value of the first singular value. This indicates that we only need to consider a handful of principal components in order to understand any changes to behaviour of the model variables when subject to parameter changes.

An easily generated plot associated with the PSS is shown in Fig. 7(b). The plots generated for SHMs are shown in Fig. 7(c). Part (c), top panel indicates that the main changes are associated with the first three PCs. We see that the biggest changes are to GI mRNA and LUX protein and the main changes occur around *t*=9. By inspecting the PSS we see that the main parameters causing a change in the direction corresponding to PC 1 (the red bars) are *n*12, *m*18 and *p*11. We also see that 11 most sensitive parameters mainly move the solution in the direction of PC1 but that the 13th (g1) moves the systems in the orthogonal direction given by PC2.

**Fig. 8:**
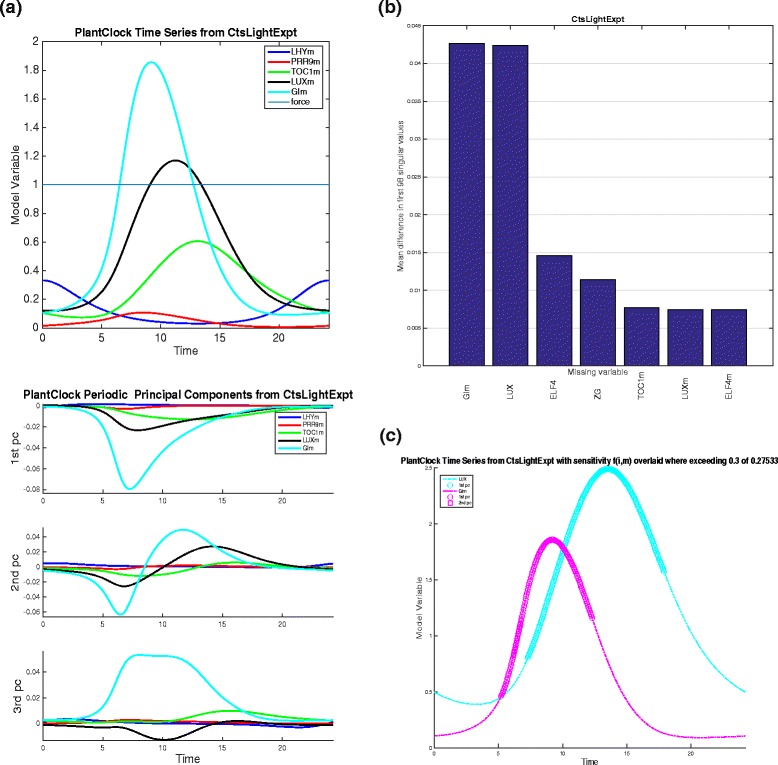
Principal Components, Singular Value Analysis plot and Time Series with Sensitivity plot. **a** Time series of several components of the plant clock model with constant light forcing (top panel) and first three principal components *U*_1_, *U*_2_ and *U*_3_ plotted for the variables selected (bottom panel). **b** Singular Value Analysis plot showing the effect on the singular values when removing one variable from the analysis in turn. Only 8 variables with the highest mean difference in the singular values from the original set of singular values are plotted. **c** Time Series with Sensitivity plot where only trajectories with the times marked when *f*_*i,m*_(*t*) for all *i,m* is larger than 30 % of the maximum value

The top three principal components, *U*_1_, *U*_2_ and *U*_3_ are shown in Fig. 8(a) and they indicate that main change to the selected time series will happen to GI mRNA, LUX and TOC1 mRNA, and this will be during the time of the peaks of these time series or when the time series levels are high. This indicates that parameter changes will change the shape of the oscillations and will most likely change the phase (i.e. peak times) of the oscillations.

The plot of Sensitivity Value Analysis in Fig. 8(b) shows that removal of GI mRNA from the SVD analysis has the largest effect on the singular spectrum. This is followed by the removal of LUX protein. Note that the higher signal value is below 0.45, so even the removal of the GI mRNA from the analysis has a relatively small effect on the singular values.

In the previous section we explained that in order to understand the changes to any sensitivity coefficients ${C^{Q}_{j}}$ related to model observables, it is enough to identify all principal components (indexed by *i*), all variables (indexed by *m*) and all times (*t*_*l*_) such that either *f*_*i,m*_(*t*_*l*_) or $f^{d}_{{i,m}}(t_{l})$ have significant values. The SHM *f*_*i,m*_ for the plant clock model are shown in Fig. 7(c). The thresholds are set at appropriate values in order to make the SHM more compact. The SHM shows that GI mRNA variable plays a large role in the value of the sensitivity coefficients and that most important timing is 6 h after the peak of LHY mRNA (the time series has been originally saved and plotted so that the start of one oscillations coincides with the peak of LHYmRNA).

The SHM of $f^{d}_{i,m}$ is shown in Fig. 7(c) in top panel. This can be used to show how, the phase changes for all the maxima and minima of the model under any parameter perturbation. The maxima times can be identified by white lines and minima ones by black lines. From Fig. 7(c) bottom panel, it becomes clear that for all the higher valued derivatives $f^{d}_{i,m}$, the peaks of LUX protein and mRNA and ELF4 mRNA happen at times of high $f^{d}_{2,m}$ i.e. when the SHM are red. This indicates that these maxima will be sensitive to parameter changes (however, not necessarily too significantly, since their SHM values are still quite low). The PSS in 7(b) shows that the phases will be most sensitive to just a handful of parameters (those with highest spectrum values |*σ*_*i*_*W*_*ij*_| for parameters *k*_*j*_. In this case, these are GI transcription and translation and mRNA degradation, i.e. *n*_12_, *p*_11_ and *m*_18_, respectively.

The times of highest sensitivity are indicated on Fig. 8(c). Only values of *i* (i.e. PC index) and *m* (variable index) where *f*_*i,m*_ are higher than 30 % of the global maximum of *f*_*i,m*_(*t*) are shown. This means that only two variables are plotted and the any change to time series of these will come about during the times indicated by markers (with each marker indicating the influence of a particular PC on the trajectory). We see that both GI mRNA and LUX protein are most affected during the time of their peaks, indicating that any parameter changes will likely bring about a phase advance or delay of the two trajectories of these two variables.

**Fig. 9:**
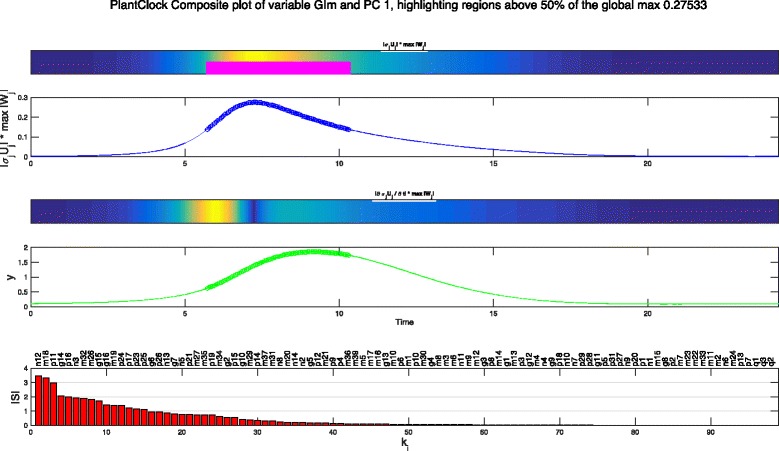
Composite plot of the plant clock model. Top three panels show the heat map and line graph *f*_1,*m*_ and the derivative heat map $f^{d}_{1,m}$where the *m* is the index of the variable GIm standing for GI mRNA. Fourth panel shows the plot of GI mRNA time series and the bottom panel shows the PSS where *S*=*σ*_1_ *W*_1*j*_ for all model parameters, *k*_*j*_, ranked in order of decreasing value

Since it is clear that the first PC indicates the largest change to the model trajectories, and all the current analysis has indicated that GI mRNA as a variable that will be most affected by any parameter changes, we can also plot the a composite plots just looking at this specific variable and the first PC. Figure 9 shows the composite plot that combines the SHMs, as well as the plot of the GI mRNA time series and the PSS for the first principal component. The analyses show that the levels when GI mRNA is rising and peaking it is most amenable to change under parameter perturbations. Again, greatest sensitivity will come from the parameter identified in the earlier PSS plot, but what is striking is that no more than 20-odd parameters out of 98 will have any bearing on this sensitivity.

**Fig. 10:**
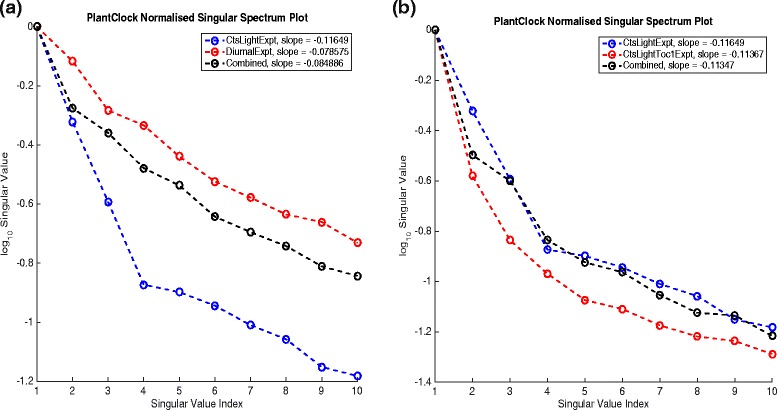
Singular value spectrum plots for different experimental combinations. **a** The singular value plot of the combined constant light and 12L:12D experiment and **b** combined constant light and *toc1* mutant constant light experiment

We can also combine different experiments together and use this functionality to see the difference in the singular values and the flexibility of the model. Addition of the the 12L:12D experiment to the continuous light experiment has different consequences to the addition of the experiment for *toc1* mutation in constant light, Fig. 10. Note that *toc1* mutant model is made by turning the translation of the TOC1 protein off (i.e. parameter *p*_4_ is set to zero). Outputs of the combined experiments are the time series of all mRNA and protein products of the combined models. The singular values of the combined experiment when the diurnal experiment is added result in a slower decay of the singular values, indicating higher flexibility to explore and optimise any of the potentially ’badly fitted’ model time series. In the case of the addition of the *toc1* mutant experiment, the opposite is the case.

## Conclusions

Here we have introduced PeTTSy (Perturbation Theory Toolbox for Systems), a free MATLAB based toolbox for analysis of large and complex biological models. PeTTSy performs simulation and analysis of models subject to a variety of conditions. It also allows experimental optimisation of complex combined experiments. PeTTSy examines sensitivity analysis of the models in a system-global setting and provides a unique set of tools, making it a valuable addition to the existing suite of sensitivity analysis toolboxes. As such PeTTSy will have broad applicability to biologists, modellers and systems biologists.

## Availability and requirements

PeTTSy can be downloaded free of charge under the terms of the GNU public license (http://www.gnu.org/licenses/gpl-3.0.en.html) from the Warwick Systems Biology Centre Software downloads page at http://go.warwick.ac.uk/systemsbiology/software. The only requirement is MATLAB, and it will run on any platform supported by MATLAB. There are though two optional dependencies. To import models from and export to SBML format requires the SBML Toolbox for MATLAB, available form http://sbml.org/Software. To use CVode in addition to MATLABŠs built in ODE solvers requires the Sundials MATLAB interface, SundialsTB, available from http://computation.llnl.gov/casc/sundials/main.html. PeTTSy uses two further pieces of third party software that come as part of the package and do not need to be installed by the user. These are SnuggleTex from the School of Physics and Astronomy, University of Edinburgh (http://www2.ph.ed.ac.uk/snuggletex), which is used to generate MathML in the export of models to SBML format, and the file findjobj.m, by Yair Altman (http://undocumentedmatlab.com) which is used in the construction of parts of the user interface. We thank all the authors of these software packages for making them available and acknowledge their contribution.

## Additional file

Additional file 1 This PDF includes the derivation of period derivatives, phase derivatives, phase infinitesimal response curves and describes the projection of the solution derivative onto rotational and amplitude variations (for the Amplitude/Phase Derivatives Scatterplot).

## References

[1] Guckenheimer J, Holmes P (1983). Nonlinear Oscillations, Dynamical Systems, and Bifurcations of Vector Fields.

[2] Hartman P (1964). Ordinary Differential Equations.

[3] Ermentrout B (2002). Simulating, Analyzing, and Animating Dynamical Systems: A Guide to XPPAUT for Researchers and Students.

[4] Maiwald T, Timmer J (2008). Dynamical modeling and multi-experiment fitting with PottersWheel. Bioinformatics.

[5] Rodriguez-Fernandez M, Banga JR (2010). SensSB: a software toolbox for the development and sensitivity analysis of systems biology models. Bioinformatics.

[6] Perumal TM, Gunawan R (2014). pathPSA: a dynamical pathway-based parametric sensitivity analysis. Ind Eng Chem Res.

[7] Zi Z, Zheng Y, Rundell AE, Klipp E (2008). SBML-SAT: a systems biology markup language (SBML) based sensitivity analysis tool. BMC Bioinformatics.

[8] Rand DA (2008). Mapping the global sensitivity of cellular network dynamics: Sensitivity heat maps and a global summation law. J R Soc Interface.

[9] Rand DA, Peixoto M, Pinto AA, Rand DA (2010). Network control analysis for time-dependent dynamical states. Dynamics, Games and Science I.

[10] Gould PD, Ugarte N, Domijan M, Costa M, Foreman J, MacGregor D, Rose K, Griffiths J, Millar AJ, Finkenstädt B, Penfield S, Rand DA, Halliday KJ, Hall AJW. Network balance via CRY signalling controls the *Arabidopsis* circadian clock over ambient temperatures. Mol Syst Biol. 2013;9(650). 10.1038/msb.2013.7 PMC3619941 23511208

[11] Domijan M, Rand DA (2010). Balance equations can buffer noisy and sustained environmental perturbations of circadian clocks. J R Soc Interface Focus.

[12] Schmidt H, Jirstrand M (2006). Systems biology toolbox for MATLAB: a computational platform for research in systems biology. Bioinformatics.

[13] Baumuratova T, Dobre S, Bastogne T, Sauter T (2013). Switch of sensitivity dynamics revealed with DyGloSA toolbox for dynamical global sensitivity analysis as an early warning for system’s critical transition. PloS ONE.

[14] Balsa-Canto E, Banga RJ (2011). AMIGO, a toolbox for advanced model identification in systems biology using global optimization. Bioinformatics.

[15] Raue A, Steiert B, Schelker M, Kreutz C, Maiwald T, Hass H, Vanlier J, Tönsing C, Adlung L, Engesser R, Mader W, Heinemann T, Hasenauer J, Schilling M, Höfer T, Klipp E, Theis F, Klingmüller U, Schöberl B, Timmer J (2015). Data2Dynamics: a modeling environment tailored to parameter estimation in dynamical systems. Bioinformatics.

[16] Hindmarsh AC, Brown PN, Grant KE, Lee SL, Serban R, Shumaker DE, Woodward CS (2005). SUNDIALS: Suite of nonlinear and differential/algebraic equation solvers. ACM T Math Softw.

[17] Rand DA, Shulgin BV, Salazar D, Millar AJ (2004). Design principles underlying circadian clocks. J R Soc Interface.

[18] Pokhilko A, Fernández AP, Edwards KD, Southern MM, Halliday KJ, Millar AJ (2012). The clock gene circuit in *Arabidopsis* includes a repressilator with additional feedback loops. Mol Syst Biol.

[19] Ashall L, Horton CA, Nelson DE, Paszek P, Harper CV, Sillitoe K, Ryan S, Spiller DG, Unitt JF, Broomhead DS, Kell DB, Rand DA, Sée V, White MR (2009). Pulsatile stimulation determines timing and specificity of NF-kappaB-dependent transcription. Science.

[20] Leloup JC, Gonze D, Goldbeter A (1999). Limit cycle models for circadian rhythms based on transcriptional regulation in *Drosophila* and *Neurospora*. J Biol Rhythms.

[21] Ruoff P (1992). Introducing temperature-compensation in any reaction kinetic oscillator model. J Interdiscipl Cycle Res.

