# Supplementary material for: PeTTSy: a computational tool for perturbation analysis of complex systems biology models
Source: BMC Bioinformatics. 2016 Mar 10;17:124. doi: 10.1186/s12859-016-0972-2 (PMC4785672; doi:10.1186/s12859-016-0972-2)
Supplement: Additional file 1 — This PDF includes the derivation of period derivatives, phase derivatives, phase infinitesimal response curves and describes the projection of the solution derivative onto rotational and amplitude variations (for the Amplitude/Phase Derivatives Scatterplot). [file 12859_2016_972_MOESM1_ESM.pdf]

Supplementary Information for "PeTSSy : a computational tool for perturbation analysis of complex systems biology models"

Mirela Domijan, Paul E. Brown, Boris Shulgin and David A. Rand

November 3, 2015

1 Period derivatives

Below we give the description of the period derivatives from Subsection 2.2.2 of the Main Text, following the derivation given in (1). Let $Y(t) = Y(t, x_0, k)$ be the solution of a matrix variation equation

$$\dot{x} = f(x, k), \quad \dot{Y} = J(t) \cdot Y, \tag{1}$$

with initial condition $x(0) = x_0, Y(0) = I_n$. Here $Y(t)$ is an $n \times n$ matrix and $J(t) = J(t, x, k)$ is the Jacobian matrix of the partial derivatives $\partial f_i / \partial x_j$ evaluated at x and k. Let τ denote the period of the periodic orbit $x = g(t, x, k)$ where τ_0 is the period of the solution at $k = k_0$ i.e. the initial parameter value. If the parameters k are changed from k_0 by $\delta k = (\delta k_1, \ldots, \delta k_s)$, then the change to period τ is

$$\partial \tau = \sum_i \partial k_i \int_0^\tau f_{k_i,\tau}(s) ds + O(||\partial k||^2) \tag{2}$$

where $f_{k_i,\tau}(s)$ are the infinitesimal response curves

$$f_{k_i,\tau}(s) = \pi_1 (Y(\tau_0) - diag([0, I_{n-1}]))^{-1} Y(\tau_0) Y(s)^{-1} h_i(s) \tag{3}$$

with $h_i(s)$ denoting the vector $\partial f / \partial k_i$ evaluated at $x = g(t, x, k_0)$ and $\pi_1(x_1, \ldots, x_n) = x_1$.

2 Phase derivatives

The description of the phase derivatives from Subsection 2.2.3 of the Main Text follows from (2). Let ϕ be the phase (maximum or minimum) of the solution $g_m(t, x, k)$. In (2) it is shown that

$$\frac{\partial \phi}{\partial k_j} = -\left(\frac{\partial \dot{g}_m}{\partial k_j}(\phi) \right) / \ddot{g}_m(\phi) \tag{4}$$

and this is in fact,

$$\frac{\partial\phi}{\partial k_j} = -\left(J_m(\phi)\frac{\partial g}{\partial k_j}(\phi) + \frac{\partial f_m}{\partial k_j}(\phi)\right) / \left(J_m(\phi)\frac{\partial g}{\partial t}(\phi)\right) \tag{5}$$

where $J_m(\phi)$ is the m-th row Jacobian evaluated at the phase of interest, corresponding to the the partial derivatives $\partial f_m/\partial x_j$, $j = 1, \ldots, n$.

3 Phase Infinitesimal Response Curves

Here we give description of the phase infinitesimal response curves (phase IRCs) from Subsection 2.2.5 of the Main Text. Consider a forced system of period τ described by the ODE model in Section 2.2.1 of the Main Text. Assume that any parameter k_j can be perturbed periodically, namely $k_j = k_j^0 + \Delta_j\alpha(s)$ where $\alpha(s) = 1$ for $s \in [\phi_1, \phi_2]$ and $\alpha(s) = 0$ for $s \in [0, \tau]\setminus[\phi_1, \phi_2]$ with ϕ_1 and ϕ_2 denoting the start and the end time of the perturbation and $\Delta_j \in \mathbb{R}$ denoting the size. The ODE model in Section 2.2.1 can be rewritten as

$$\frac{dx}{dt} = F(t, x, k, \Delta_j) \tag{6}$$

with $F = f(t, x, k_1, \ldots, k_j^0 + \Delta_j\alpha(s), k_{j+1}, \ldots, k_s)$.

Let $x = g(t, k, \Delta_j)$ be the periodic solution of (6). Then $y(t) = \frac{\partial g}{\partial \Delta_j}(t)$ is the periodic solution of

$$\dot{y} = J(t)y + K(t) \tag{7}$$

where $J(t)$ is the Jacobian matrix Df_x evaluated at $x = g(t, k, 0)$ and $K(t) = \frac{\partial F}{\partial \Delta_j}(t) = \frac{\partial f}{\partial k_j}(t)\alpha(t)$ with $\frac{\partial f}{\partial k_j}(t) = \frac{\partial f}{\partial k_j}(t, g(t, k, 0))$. Let $X(s, t)$ denote the solution of $\frac{dX}{dt} = J(t)X$ with $X(s, s) = I$ where I is the identity matrix and let us introduce the notation $X_t = X(t, t+\tau)$. It is easy to check that since y is a periodic solution of (7), it takes the form

$$y(t) = (I - X_t)^{-1}\int_t^{t+\tau} X(s, t+\tau)\frac{\partial f}{\partial k_j}(s)\alpha(s)ds. \tag{8}$$

Now, let $\phi_m = \phi_m(k, \Delta_j)$ be the time when g_m has a maximum (or minimum), namely, $\dot{g}_m(\phi_m(k), k, \Delta_j) = 0$. In (2) it is shown that

$$\frac{\partial \phi_m}{\partial \Delta_j} \quad = \quad -\left(\frac{\partial}{\partial t}\frac{\partial g_m(t)}{\partial \Delta_j}\bigg|_{t=\phi_m}\right) / \ddot{g}_m(\phi_m) \tag{9}$$

and together with (7) and (8) it follows that

$$\frac{\partial}{\partial t}\frac{\partial g_m}{\partial \Delta_j}(t) \quad = \quad \frac{\partial f_m}{\partial k_j}(t)\alpha(t) + J_m(t)\frac{\partial g_m}{\partial \Delta_j}(t) \tag{10}$$

and

$$\frac{\partial \phi_m}{\partial \Delta_j} = \frac{-1}{\ddot{g}_m(\phi_m)} \left(\frac{\partial f_m}{\partial k_j}(\phi_m)\alpha(\phi_m) + J_m(\phi_m)(I - X_{\phi_m})^{-1} \int_{\phi_m}^{\phi_m+\tau} X(s, \phi_m + \tau) \frac{\partial f}{\partial k_j}(s)\alpha(s)ds \right)$$

where J_m is the m-th row of the Jacobian matrix $J(t)$. Note that in the software we refer to the first term of the sum in (11) as $\Delta\phi$ (Part II) and the second term of the sum (i.e. the integral) as Part I.

4 Projection onto rotational and amplitude variations

Suppose that $g(t)$ is our periodic solution of interest and consider an infinitesimal perturbation δg of it. If we move the phase of g by an amount α we get the time series $\tilde{g}(t)g(t + \alpha)$. The derivative of this with respect α at $\alpha = 0$ is given by

$$\frac{d}{dt}\tilde{g}|_{\alpha=0} = \dot{g}(t).$$

Thus the rotational part of δg is given by the inner product of δg and the unit vector $n(t) = \dot{g}/||\dot{g}||$ in the direction $\dot{g}$ i.e.

$$\tau^{-1} \int < \delta g(t), n(t) > \, dt$$

where the integral is over one period and the period is τ. Here $||\delta g|| = \tau^{-1} \int ||g(t)||^2$. Note that n is the vector R_m in the main text.

Similarly an amplitude change to g is given by $\tilde{g} = (1 + \alpha)g$ and thus the infinitesimal amplitude is given by $(d/d\alpha)|_{\alpha=0}\tilde{g} = g$. Therefore the amplitude part of δg is given by the inner product of δg and the unit vector $g/||g||$ (vector A_m in the main text).

References

[1] Rand, D . A. and Shulgin, B. V. and Salazar, D. and Millar, A. J. (2004) Design principles underlying circadian clocks, *Journal of the Royal Society Interface*, **1**, 119-130.

[2] Rand, D. A. (2008) Mapping the global sensitivity of cellular network dynamics: Sensitivity heat maps and a global summation law. *J. R. Soc. Interface*, **5** S59-S69.
